# Therapeutic hyperthermia for the treatment of infection—a narrative review

**DOI:** 10.3389/fphys.2023.1215686

**Published:** 2023-07-26

**Authors:** Andrej Markota, Žiga Kalamar, Jure Fluher, Sergej Pirkmajer

**Affiliations:** ^1^ Medical Intensive Care Unit, University Medical Centre Maribor, Maribor, Slovenia; ^2^ Faculty of Medicine, University of Maribor, Maribor, Slovenia; ^3^ Institute of Pathophysiology, Faculty of Medicine, University of Ljubljana, Ljubljana, Slovenia

**Keywords:** therapeutic hyperthermia, targeted temperature management, infection, sepsis, intensive care unit

## Abstract

Modulating body temperature, mostly through the use of antipyretics, is a commonly employed therapeutic intervention in medical practice. However, emerging evidence suggests that hyperthermia could serve as an adjuvant therapy for patients with infection. We performed a narrative review to explore the application of therapeutic hyperthermia in the treatment of infection. A number of studies have been performed in the pre-antibiotic era, enrolling patients with neurosyphilis and gonococcal infections, with reported cure rates at around 60%–80%. We have outlined the potential molecular and immunological mechanisms explaining the possible beneficial effects of therapeutic hyperthermia. For some pathogens increased temperature exerts a direct negative effect on virulence; however, it is presumed that temperature driven activation of the immune system is probably the most important factor affecting microbial viability. Lastly, we performed a review of modern-era studies where modulation of body temperature has been used as a treatment strategy. In trials of therapeutic hypothermia in patients with infection worse outcomes have been observed in the hypothermia group. Use of antipyretics has not been associated with any improvement in clinical outcomes. In modern-era therapeutic hyperthermia achieved by physical warming has been studied in one pilot trial, and better survival was observed in the hyperthermia group. To conclude, currently there is not enough data to support the use of therapeutic hyperthermia outside clinical trials; however, available studies are in favor of at least a temperature tolerance strategy for non-neurocritical patients.

## Introduction

Body temperature modulation is a common therapeutic intervention in medicine (Laupland et al., 2008). Targeted temperature management (TTM) is widely used during surgery to prevent periprocedural hypothermia (Simegn et al., 2021); however, on hospital wards and in intensive care units (ICUs), TTM is mainly intended to reduce increased body temperature (Laupland et al., 2008). The rationale for temperature reduction is based on reduced metabolic demands associated with decreased body temperature and presumed increased patient comfort (Laupland et al., 2008). Despite longstanding use of temperature modulation in clinical practice, specific target temperatures have only been defined for a small subset of patients—i.e., unconscious patients after cardiac arrest and neurocritical patients (Drewry and Mohr, 2022; Nolan et al., 2022). Optimal target temperatures for other patient groups remain uncertain.

In most patients with infection, fever is part of a normal response of the immune system. Patients with sepsis who are incapable of mounting a fever response experience increased mortality compared to patients with sepsis who can develop fever (Kushimoto et al., 2013). Increase in temperature during infection is present in organisms as diverse as humans, reptiles, insects, fish (Evans et al., 2015), and even plants (Yarwood, 1953). In the pre-antibiotic era, warming patients - therapeutic hyperthermia (TH) or pyrotherapy—was already used to treat infections (Owens, 1936; Wagner-Jauregg and Bruetsch, 1946). The advent of antibiotics and their effectiveness in treating infections has hindered the development of alternative treatment options for patients with infection (Streicher, 2021). However, the emergence of (multiple) drug resistant microorganisms and pandemics of viral infections (e.g., influenza, SARS, MERS, COVID-19) emphasize the importance of exploration of alternative infection treatment methods—ones that are not based on standard antimicrobial therapy (Herder et al., 2021). The present article describes TH as a potential additional treatment method for patients with infection.

## Methods and terminology

We performed a non-structured review of literature in the PubMed database using search terms “therapeutic hyperthermia”, “fever”, “targeted temperature management”, “hyperthermia”, hyperpyrexia”, “fever management”, “cellular mechanisms of fever”, “immunological mechanisms of fever” and various combinations of the aforementioned terms. The search for clinical studies was performed by AM, ŽK and JF, while SP and ŽK conducted the search for laboratory and immunological studies. Due to the wide scope of the review, which included data from trials conducted in both the pre-antibiotic era to modern-era studies, as well as laboratory studies and clinical trials, a structured approach was not feasible.

Throughout the text, different terms are used to describe increased body temperature and therapeutic interventions associated with body temperature, such as fever and hyperthermia, and therapeutic hyperthermia, therapeutic hypothermia and normothermia. We tried to use the most widely accepted definitions of temperature ranges, acknowledging that different temperature ranges are reported, and that body temperature depends on the location of measurement (Mackowiak et al., 1992). We use the term fever to describe elevation of body temperature ≥37.7°C associated with infection or presumed infection. We use the term hyperthermia to describe any elevation of body temperature ≥37.7°C. Terms physiological range fever or physiological range hyperthermia are used to describe elevations of body temperature between 37.7°C and 40.5°C (Mackowiak et al., 1992). Regarding therapeutic modulation of body temperature, the term therapeutic hyperthermia is used to denote artificially increased body temperature above 37.7°C. The upper range of temperature is not well defined because of the paucity of the data. The term therapeutic hypothermia is used to denote artificially decreased body temperature between 32°C and 34°C, and the term normothermia is used to denote active maintenance of body temperature between 36°C and 37°C (Nolan et al., 2022).

## Caveats and adverse effects of therapeutic hyperthermia

While this article discusses the potential beneficial effects of TH in the setting of infections, there is very little direct data from modern era demonstrating its clinical benefit in this patient cohort (Wagner-Jauregg and Bruetsch, 1946; Drewry and Mohr, 2022). Laboratory and animal studies that outline potential mechanisms of action of TH in the setting of infection have been conducted in various settings, with a significant number of studies aiming to determine potential immunomodulatory effects of TH in the setting of treatment of various malignomas (Evans et al., 2015). Additionally, adverse events also need to be considered. Upper limits of body temperature depend on the patient; in patients with an acute involvement of the central nervous system (e.g., trauma, hemorrhage, infection, diffuse ischemia after cardiac arrest…) maintenance of normothermia is usually advised (Drewry and Mohr, 2022; Nolan et al., 2022). Because of an increase in basal metabolism associated with an increase in body temperature, patients with heart failure might not adequately escalate cardiac output even in physiological fever range hyperthermia (Laupland et al., 2008). In these patients temperature tolerance could potentially be utilized, and careful clinical, hemodynamic, respiratory, and metabolic monitoring should be applied in case of physiological range fever. If the patient tolerates fever without deterioration, withholding antipyretic therapy could be considered. Body temperature above 40.5°C is usually considered as severe hyperthermia and heat stroke can develop. Heat stroke is associated with significant mortality (around 20%–40%), and symptomatic therapy (active cooling) is a mainstay of therapy (Drewry and Mohr, 2022).

Additionally, the role of whole-body hyperthermia to treat infection is presented in the text, as opposed to local heating. There is some data to support the use of local heating to promote surgical wound healing and to treat or prevent biofilm formation, using different modalities of heating, such as heating by radiofrequency, ultrasound, microwaves, and magnetic nanoparticles (Ibelli et al., 2018), however, local heating is not discussed in the following text.

## Therapeutic hyperthermia and management of fever in other settings

TH has long been used as adjuvant treatment of malignant disorders. Clinically well-established treatment regimens involve thermal therapies for local and locoregional heating, however, whole-body hyperthermia is used rarely in this setting. Direct tissue and organ heating between 39°C and 45°C is applied to induce sensitization to radio- or chemotherapy. Thermal tissue ablation, where temperatures above 50°C are used, directly targets and destroys tumor cells (Kok et al., 2020). Whole-body hyperthermia either in physiological range hyperthermia (39°C–40°C core body temperature measured in esophagus or rectum) or extreme hyperthermia (41°C–42°C core body temperature) has been studied in combination with systemic chemotherapy for treatment of metastatic solid tumors. Treatment regimens involve sedation and general anesthesia for application of radiant heat (infrared radiation) or veno-venous extracorporeal circulation heating to achieve target temperature and maintain it for the duration of the plateau phase of 60 min. Mechanisms of malignant disease inhibition are chemotherapy potentiation and neoplastic cell destruction. Phase I and II trials that have been performed for advanced colorectal adenocarcinoma, lung cancer and ovarian cancer report serious adverse effects, mostly myelosuppression that could also be attributed to concomitant chemotherapy (Hildebrandt et al., 2005; Lassche et al., 2019; Kok et al., 2020).

While specific guidelines regarding target body temperatures exist for adults with acute central nervous system involvement, such as comatose patients after cardiac arrest (Nolan et al., 2022), there are no guidelines for general patient population. For pediatric population temperature tolerance for children with fever has been accepted as a treatment strategy, where patient’s overall comfort is considered along with the temperature readings, with the possible exception of children with underlying chronic disease and critically ill children (Sullivan and Farrar, 2011).

## Therapeutic hyperthermia in the pre-antibiotic era

Mortality rates associated with infectious diseases were exceedingly high during the pre-antibiotic era (Runcie, 2015). Aside from surgical treatment of infections (e.g., drainage, necrectomy and amputations), other treatment options were severely limited, and additional treatment methods were actively researched (Runcie, 2015). TH was one of the most well researched treatment interventions aimed at curing infections (Owens, 1936; Wagner-Jauregg and Bruetsch, 1946; Daey Ouwens et al., 2017).

### Malariotherapy in the treatment of neurosyphilis

In the beginning of the 20th century, syphilis, caused by the bacterium *Treponema pallidum*, was widely spread in various stages of the disease (Daey Ouwens et al., 2017). In Europe and North America it affected around 10% of the population. Tertiary syphilis, characterized by the involvement of the central nervous system and the onset of dementia paralytica, imposed a significant public health burden on society. Approximately 25% of all new patients were admitted to psychiatric wards because of neurosyphilis (dementia paralytica), with mortality approaching 100% (Frith, 2012). The first reports on the use of TH in patients with dementia paralytica are from the beginning of the 20th century (Wagner-Jauregg and Bruetsch, 1946; Daey Ouwens et al., 2017). Due to the large number of patients a simple and reliable way of inducing hyperthermia was required (Wagner-Jauregg and Bruetsch, 1946). This was achieved by inoculation of the parasite *Plasmodium* spp., after which cyclic increases in body temperature occurred as part of malaria. Even though malaria was used to induce hyperthermia the researchers recognized the role of temperature increases *per se*, and not malaria, as crucial in the treatment of neurosyphilis (Wagner-Jauregg and Bruetsch, 1946; Daey Ouwens et al., 2017). Cure of syphilis was reported in 50%–80% of patients, and malaria would be treated with quinine. Mortality due to treatment complications was around 10%–15% (Wagner-Jauregg and Bruetsch, 1946; Daey Ouwens et al., 2017). The importance of TH in the treatment of syphilis is also evidenced by the awarding of the Nobel Prize for Medicine in 1927 to Julius Wagner-Jauregg for the discovery of the treatment of paralytic dementia by malaria inoculation (Wagner-Jauregg and Bruetsch, 1946).

### Therapeutic hyperthermia for the treatment of gonococcal infections

While malariotherapy required inoculation of a parasite to induce temperature increases to treat neurosyphilis, direct physical warming was used in the 1930s to treat infections caused by bacterium *Neisseria gonorrhoeae* (Owens, 1936). Body temperatures of around 41°C could reliably be achieved in around 1 h in specially designed hyperthermia chambers (Kettering Isotherm chamber). In a study performed by Owens, patients with different gonococcal infections were heated to a body temperature of 41°C for about 5 h. Usually, three to four hyperthermia sessions were required. Of the 100 patients who were included in the study, 64 completed the treatment and 52 were cured (81%). Twenty-four patients refused further treatment due to non-tolerance, and 12 could not continue treatment due to comorbidities (Owens, 1936). For comparison, cure rates of around 95% can be achieved with antibiotic therapy (Handsfield et al., 1991).

## Mechanisms of action of therapeutic hyperthermia

There are several potential mechanisms of action that could account for the presumed beneficial effect of hyperthermia on the treatment of infection. One such mechanism involves the direct inhibitory effect of increased temperature on microbial virulence, while others include the effects of hyperthermia on increased organ perfusion, immune system activation, and susceptibility to antibiotic therapy (Mackowiak et al., 1982; Evans et al., 2015; Gao et al., 2017).

### Effect of increased temperature on microbial virulence

Direct anti-pathogen effects of physiological range hyperthermia are based on an upper temperature limit at which microbial replication and viability are impaired (maximum tolerated temperature). Attenuation of either replication capability or viability are beneficial to the host because the number of pathogens is decreased (Casadevall, 2016). Body temperature (or elevation of body temperature) can thus provide an environmental thermal exclusion zone against susceptible microbes. Many bacterial species are restricted at temperatures above 25°C, which makes human infection very unlikely (Stuart-Smith et al., 2015). Conversely, human (and mammalian) pathogens have adapted to higher temperatures enabling infection.

**FIGURE 1:**
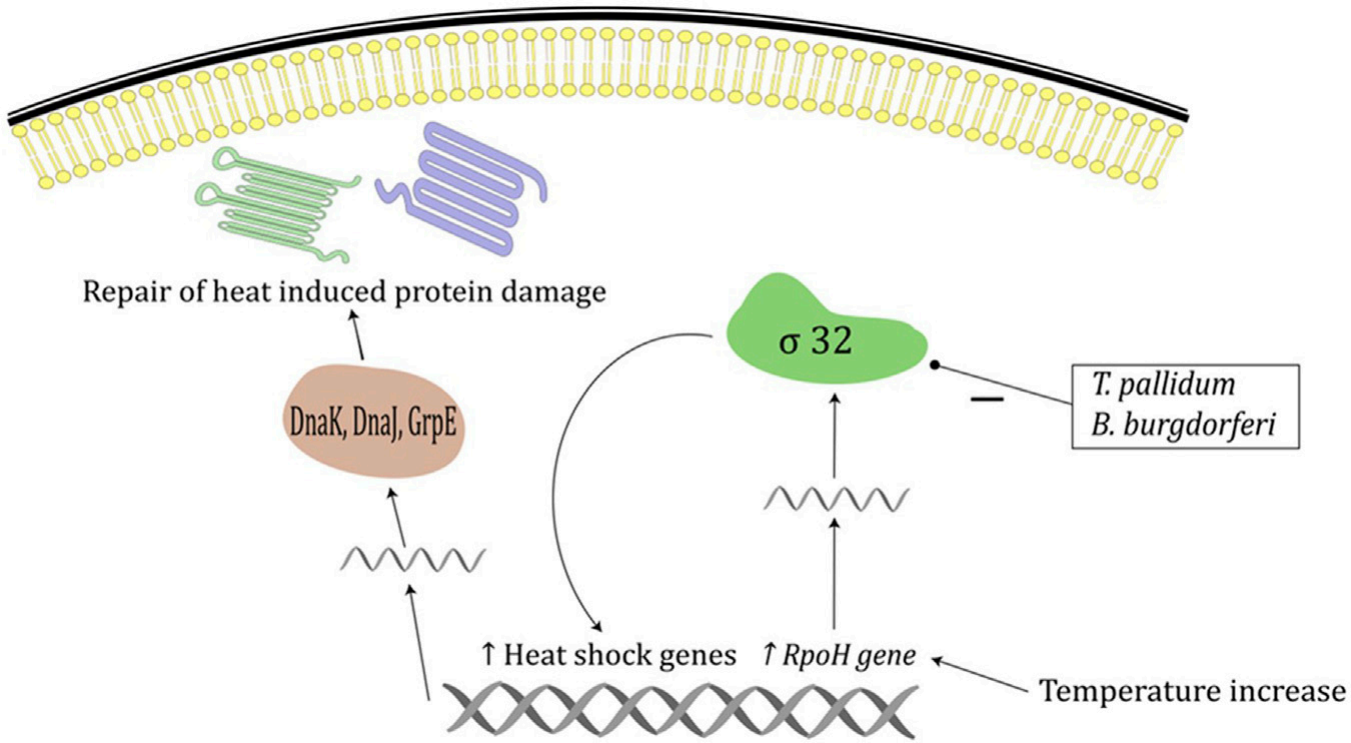
Heat shock response in prokaryotes. Temperature increase induces increased expression of RpoH gene and increased *σ* 32 levels which increases expression of heat shock proteins including DnaK, DnaJ and GrpE chaperone system. Chaperone systems are responsible for repair of heat induced protein damage. *Treponema pallidum* and *B. burgdoferi* lack factor *σ* 32.

Several mechanisms contribute to thermal restriction, and it affects bacteria, viruses, and fungi. Although prokaryotes in general are viable over a wide spectrum of environmental conditions, most bacterial species causing human infection have a specific temperature range in which they thrive. Bacterium *T. pallidum* grows optimally at a temperature range 32°C–36°C, while exposure to 42°C inactivates it completely (Porcella and Schwan, 2001). This is probably explained by the lack of *σ* factor 32 protein in *T. pallidum*, which serves as a primary mediator of the heat-shock response (Figure 1). Similarly, while bacterium *B. burgdorferi* grows optimally at 37°C, its growth rate slows at 39°C, while exposure to 41°C kills all the bacteria in the medium, which could also be explained by the lack of *σ* factor 32 in *Borrelia burgdorferi* (Figure 1) (Porcella and Schwan, 2001). The bacterium *N. gonorrhoeae* responds to heat shock by the activation of alternative *σ* factor 32 pathway which in turn downregulates a large number of genes involved in protein biosynthesis and maintenance of protein homeostasis, which could contribute to susceptibility to increased temperature (Gunesekere et al., 2006).

**FIGURE 2:**
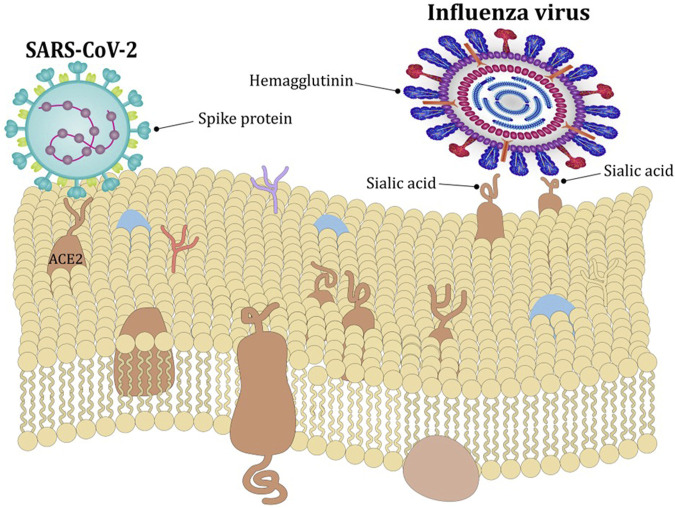
Effect of temperature on viral entry. The expression of viral hemagglutin is greater around 33°C compared to higher temperatures which facilitates membrane fusion and infection with influenza virus. The interaction between Spike glycoprotein and ACE2 receptor which facilitates entry of SARS-CoV-2 is enhanced at around 37°C and restricted at temperatures around 40°C.

Virulence of different isolates of human rhinoviruses is attenuated by increasing temperatures, with faster replication rates in the temperature range typically found in the nasal cavity (33°C–35°C) compared to core body temperature (around 37°C) (Foxman et al., 2015). Similarly, virulence of influenza B virus is greater around 33°C compared to higher temperatures. The mechanism is increased expression of viral hemagglutinin at 33°C facilitating membrane fusion and infection (Figure 2) (Laporte et al., 2019). Temperature also affects the virulence of SARS-CoV-2, which attaches to respiratory tract cells by binding the Spike S) glycoprotein to the angiotensin-converting enzyme 2 (ACE2). This interaction is enhanced at temperatures around 37°C and restricted at temperatures around 40°C (Figure 2) (Zhou et al., 2021). Once infected, intracellular viral replication is also restrained by higher temperatures via decreased messenger-RNA processing (Herder et al., 2021).

Fungus *Candida albicans* responds to elevation of temperature from 37°C to 39°C by downregulating genes involved in cellular wall metabolism and maintenance of cell wall integrity. Also, at a temperature of 42°C biofilm formation does occur, but its bulk, thickness, and metabolic activity are restricted compared to 37°C (Pumeesat et al., 2017). Similarly, growth of the fungus *Aspergillus niger* is restricted by temperatures of 40°C and electron microscopy demonstrated disintegration of mitochondria with a six to tenfold increase in oxidatively damaged proteins (Abrashev et al., 2014).

### Effect of increased temperature on organ perfusion

Higher body temperature is associated with increased metabolic rate, with approximately 10% increase in oxygen consumption for every 1°C increase in body temperature (Kluger, 1979). Increased metabolic rate requires an increase in cardiac output and peripheral tissue perfusion via peripheral vasodilation. It is speculated that increased perfusion correlates with more rapid hemodynamic stabilization (Gao et al., 2017). In a sheep model of peritonitis and septic shock, the sheep in the high temperature group developed higher oxygenation index, lower lactic acid and longer survival times compared to sheep in the lower temperature group (Su et al., 2005). Similar findings were observed by Gao et al. (2017). They performed a prospective study in adult patients with septic shock and body temperature ≥39°C. Patients were randomized to two groups: low temperature group (target core body temperature 36.5°C–38°C) and high temperature group (target core body temperature 38.5°C–39.5°C), and temperature was maintained within target range with antipyretic medication and intravascular catheters. Lactate concentration and noradrenaline requirement were significantly lower in the high temperature group, implying more rapid hemodynamic stabilization. Also, heart rate, stroke volume and cardiac output were significantly higher in the high temperature group, and systemic vascular resistance index was significantly lower in the high temperature group (Gao et al., 2017).

### Effect of increased temperature on immune system activation

Immune system is activated both in the context of fever and in exogenous hyperthermia, such as heat stroke (Evans et al., 2015). Activation of the immune system associated with fever is probably the most important inhibitory effect on microbial viability. It is proportional to the increase of body temperature within the physiological range of fever. Both the innate and the adaptive immune system are activated, and a number of changes can be observed, namely,: enhanced innate immunity, including increased phagocytic activity of leukocytes, increased mobility of leukocytes, and expression of specific interleukins (Roberts and Steigbigel, 1977; Evans et al., 2015).

**FIGURE 3:**
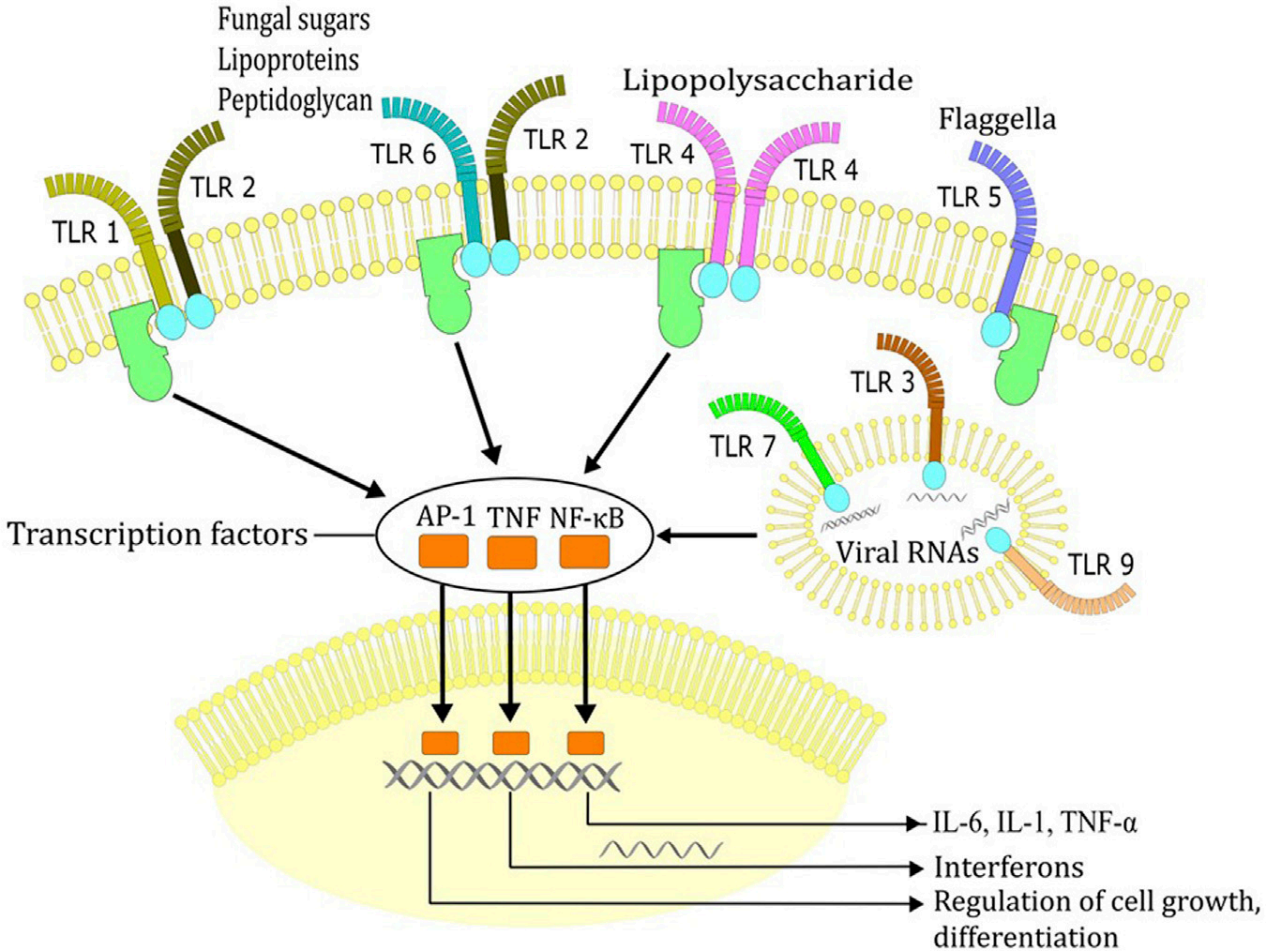
Recognition of pathogen associated molecular patterns (e.g., viral RNA, lipopolysaccharide, fungal sugars, flagella) by Toll-like receptors (TLR). Recognition of these molecules by TLRs triggers signal transduction cascades that ultimately induce the expression of pro-inflammatory cytokines, interferons or IFN-inducible genes which dictate the outcome of innate immune responses.

Mechanisms of fever are covered in excellent reviews by Evans et al. (2015) and Dantzer (2018), therefore just a brief overview is provided here. Immune system activation in infection begins with the recognition of pathogen associated molecular patterns (e.g., viral RNA, lipopolysaccharide (LPS), or fungal sugars) by pathogen recognition receptors (e.g., Toll-like receptors) on cells that form innate immune system (e.g., macrophages, neutrophils, or dendritic cells) (Figure 3). In response to invasion of pathogens, immune cells produce autacoids, such as prostaglandin E2 (PGE2), as well as cytokines, such as IL-1 and IL-6. When PGE2, IL-1 and IL-6 reach the brain they stimulate further local production of PGE2 and IL-6, thus leading to physiological responses that reduce the loss and increase the production of heat (Evans et al., 2015; Dantzer, 2018). Peripheral activation of vagal afferents by PGE2 may present an additional mechanism by which thermoregulatory responses are elicited to produce fever (Dantzer, 2018). The heat loss is suppressed by reduction in cutaneous blood flow and by an increase in sweating, while the heat production is increased by contractions of muscle fibres (shivering) and activation of the brown adipose tissue (Evans et al., 2015; Dantzer, 2018). However, production of pyrogenic cytokines, such as IL-6, is also augmented by elevation of core body temperature due to exertion and/or high external temperature (Welc et al., 2012). While secretion of cytokines may contribute to pathogenesis and poor outcome of exertional heat injury (Robins et al., 1995; Lu et al., 2004; Lim et al., 2007; Welc et al., 2012), fever and the attendant activation of the immune system likely provide evolutionary advantage in defence against microbes. Consistent with this view, both endothermic (e.g., humans), as well as ectothermic (e.g., reptiles) organisms change behavioral patterns in order to increase their body temperature (e.g., humans with additional clothing, reptiles with sun exposure) (Bicego et al., 2007).

Immune responses to exogenous hyperthermia due to passive heating need to be considered briefly for two reasons. First, as highlighted before (Evans et al., 2015) experimental hyperthermia has been used to study immunological effects of fever-range temperatures. Second, fever and heat stroke share some of the immune responses despite having fundamentally different initiating causes. In contrast to fever, where an increase in body temperature is driven by the immune system, heat stroke occurs due to inability of the body to dissipate heat, usually due to exposure to hot and humid environment, especially in the context of physical exertion (Leon and Helwig, 2010; Périard et al., 2021; Cramer et al., 2022; Schlader et al., 2022). Under these conditions, the increase in body temperature elicits physiological reactions to oppose further heating and reduce body temperature, including an increase in the cutaneous blood flow and sweating. On the other hand, due to vasoconstriction in the gastrointestinal tract, the gut epithelium becomes leaky in heat stroke patients, leading to penetration of LPS into the blood (Leon and Helwig, 2010; Schlader et al., 2022). Once in the circulation, LPS induces cytokine release, which may contribute to hyperthermia. While increased levels of plasma levels of TNF-α, IL-1α, and IL-6 were observed in heat stroke patients (Bouchama et al., 1991; Hammami et al., 1997) blockade of IL-1β action by the endogenous IL-1 receptor antagonist protein (aka IL-1ra) in animal experiments improved survival (Chiu et al., 1996), indicating an important role for IL-1 in pathogenesis of heat stroke. Moreover, cytokine secretion may also lead to fever in heat stroke patients (Leon and Helwig, 2010), highlighting that mechanisms of heat stroke and fever overlap despite having distinct etiopathogenesis.

Clearly, when considering the effect of body temperature on the immune system several aspects need to be considered. First, activation of the immune system may be the primary event, which precedes an increase in the body temperature, or a secondary event, which occurs after body temperature has already increased. Second, the body temperature may directly modulate the function of immune cells and organs. Third, thermoregulatory physiological reactions, such as altered vascular function, which contribute to or occur in response to increased body temperature may indirectly impact the immune system. Finally, as outlined above, the same mediators, such as IL-1 and IL-6, which affect both immune and thermoregulatory functions, are involved in orchestrating response to infectious agents as well as to increased body temperature in the absence of infection. Under *in vivo* conditions, where an integrated response takes place, it might therefore be difficult to dissect direct effects of temperature on the immune system, especially in the context of fever. This is one of the reasons why experimental hyperthermia was used to study effects of fever-range temperatures (Evans et al., 2015). Conversely, while under *in vitro* conditions biological effects of temperature can be examined independently from systemic reactions, not all aspects of the immune response, which requires integrated action by many types of cells and organs, can be assessed in this way. With these limitations in mind, some of the studies which used different approaches to investigate effects of temperature on immune system are highlighted in continuation.

A number of mechanisms have been described that contribute to enhanced innate immunity. Physiological range increase in body temperature (i.e., 1°C–4°C above normal temperature in mammals) increases migration of neutrophils to peripheral tissues (Evans et al., 2015). Neutrophil function such as bactericidal activity against some pathogens, is also enhanced by fever, and conversely, neutrophil function is impaired by further elevation of body temperatures above physiological febrile range of temperatures (Ostberg and Repasky, 2000). Neutrophil activation may be in some part driven by temperature dependent release of granulocyte colony-stimulating factor (G-CSF), which in turn is driven by enhanced production of IL-17, IL-1α and IL-1β (Capitano et al., 2012). Fever range temperatures upregulate the activity and localized recruitment of natural killer lymphocytes. This is mediated by increased clustering of NKG2D receptors on natural killer cells and increased expression of MHC class I-related chain A (MICA), a NKG2D ligand, which is absent on the surface of normal cells, but is expressed on the surface of infected, stressed or transformed cells (Ostberg et al., 2007). Macrophages are activated by temperature dependent upregulation of heat shock protein 70 (HSP70). HSP70 induces intracellular changes in macrophages but is also released into extracellularly. Extracellular HSP70 can activate multiple receptors on macrophages and dendritic cells (Hatzfeld-Charbonnier et al., 2007). Increased temperature upregulates the expression of receptors on macrophages and dendritic cells, enhancing migration and phagocytosis. Furthermore, increased temperatures enhance the ability of macrophages and dendritic cells to activate T cells and promotes differentiation of T cell line (Basu et al., 2001). Migration of antigen-presenting cells to draining lymph nodes is also accelerated by increased temperatures via temperature-dependent increase in sensitivity of chemokine receptor type 7 (Vardam et al., 2007).

The development of adaptive immunity depends on high circulation rate of lymphocytes through the lymphatic tissue. Circulating lymphocytes need to bind to specialized endothelial zones where lymphocyte entry to lymph nodes is enabled—high endothelial venules. Increased temperatures induce an approximately 2-fold increase in the ability of lymphocytes T or B to bind to high endothelial zones (Wang et al., 1998). Increased binding capability is mediated through the increased binding activity of L-selectin and α4β7 integrin without increasing the number of these molecules. Increased binding capability is temperature dependent—it occurs in passive heating and in lipopolysaccharide induced fever (Evans et al., 2000). Increased binding capability is time dependent and requires prolonged (at least 6 h) duration of temperature in the range of physiological fever (Mace et al., 2012).

The ability of lymphocytes to differentiate is also enhanced by fever range temperatures. Increased temperature (39°C vs. 37°C vs. 33°C), which correlates with temperature dependent activation of protein kinase C β, stimulates CD8^+^ lymphocytes T and enhances production of IFN-γ (O'Sullivan et al., 2021). Moreover, exposure of CD8^+^ T cells to high temperature (39°C) *ex vivo* enhances their antitumor activity *in vivo* (Umar et al., 2020), indicating fever may enhance their effector functions also against the invading microbes. Interestingly, CD4^+^ cells exposed to 39°C show switch towards the Th2 phenotype and reduced Th1 commitment (Wang et al., 2020). This effect was dependent on heat-sensitive transient receptor potential vanilloid channels (TRPV) TRPV1 and TRPV4 (Ostberg et al., 2007) which suggests a mechanism by which fever could affect the Th1/Th2 balance, thus modulating the immune response. Recent data also shows that temperatures in the febrile range promote differentiation of Th17 cells, which are important players in host defence, towards a more pro-inflammatory phenotype (Wang et al., 2020).

In summary, increased body temperature in the context of fever apparently promotes various aspects of immune function, thus enhancing its protective effects against infectious agents and facilitating the resolution of infection (Evans et al., 2015). However, on the other hand, heat stroke demonstrates that the same or similar mechanisms, such as cytokine secretion, may become counterproductive and cause additional complications, thereby reducing the chances of survival (Leon and Helwig, 2010). Since hyperthermia is a continuum, the extent of body temperature increase *per se* may determine whether protective or deleterious effects prevail. Alternatively, temperature-dependent activation of immune system in fever and heat stroke might be fundamentally different. These interesting possibilities will have to be dissected by research in the future.

### Effect of increased temperature on susceptibility to antibiotic therapy

Increased temperatures can increase both *in vitro* and *in vivo* effectiveness of some antibiotics. Minimal inhibitory concentrations are decreased in physiological range hyperthermia (41.5°C vs. 40°C vs. 38.5°C vs. 37°C vs. 35°C) for numerous antimicrobial agents, including penicillins, cephalosporins, aminoglycosides, tetracycline, trimethoprim-sulfamethoxazole, clindamycin, and erythromycin (Mackowiak et al., 1982). The effect of decreased minimal inhibitory concentrations has been observed in numerous bacterial species, including Gram-positive cocci and Gram-negative bacteria. At the temperature 41.5°C minimal inhibitory concentrations were at least 16-times lower compared to 35°C (Mackowiak et al., 1982). Similarly, serum bactericidal activity at 42°C was increased compared to 32°C, and binding to serum proteins did not decrease the effect of temperature on bacterial killing (Oesterreicher et al., 2019). Also, for some antibiotics increased plasma concentrations were observed in fever range temperatures. In rats, higher plasma concentrations of ertapenem were observed in fever compared to normothermia (Boulamery et al., 2008). Similarly, in humans higher maximum serum concentrations of ciprofloxacin and cefazolin were observed during fever (Beovic et al., 1999). Increased bactericidal effects of daptomycin, vancomycin, tigecycline, fosfomycin and cefamandole were also observed in the setting of bacterial biofilms, which present an important bacterial defensive mechanism. Increasing ambient temperature from 35°C–45°C reduces the thickness of staphylococcal biofilms, with a more pronounced effect observed for daptomycin and cefamandole (Hajdu et al., 2010). Similar effects were observed in *Pseudomonas aeruginosa* biofilms exposed to ciprofloxacin (Aljaafari et al., 2021).

## Current state of therapeutic hyperthermia

There is very little direct data on the use of TH from modern era clinical studies. However, a number of studies have been performed in the field of temperature modulation in patients with sepsis, ranging from therapeutic hypothermia to avoidance of fever to therapeutic hyperthermia (Table 1) (Mourvillier et al., 2013; Young et al., 2015; Itenov et al., 2018; Drewry et al., 2022; Markota et al., 2022). The direct mechanisms of action of temperature modulation are mostly not discussed or elaborated on in clinical studies; however, the effects or associations of temperature modulation are presented as different clinical outcomes depending on various target temperatures. Also, various methods have been used to modulate temperature, which makes any generalization difficult at this time.

**TABLE 1:** Overview of the studies presented in the text.

Study design	Patients	Number of patients	Therapeutic intervention	Findings	References
Prospective uncontrolled interventional trial	Gonococcal infections	100	Hyperthermia chambers, warming to 41°C for 5 h, 3–4 sessions	81% cure rate in patients who completed the treatment; 24% did not complete due to non-tolerance, 12% did not complete due to comorbidities	Owens
					(Owens, 1936)
Case series	Community-acquired bacterial meningitis	10	Induced hypothermia (32°C–34°C).	6 out of 10 patients survived	Lepur et al. (2011)
Multicenter RCT	Community-acquired bacterial meningitis	98	Hypothermia group (32°C–34°C) vs standard care	Trial stopped early because of higher mortality in the hypothermia group (51% vs. 31%, *p* = 0.04)	Mourvillier et al. (2013)
Multicenter RCT	Severe sepsis or septic shock	436	Hypothermia group (32°C–34°C) vs standard care	Trial stopped early for futility. Higher mortality in the hypothermia group (44.2% vs. 35.8%, *p* = 0.07)	Itenov et al. (2018)
Multicenter RCT	Known or suspected infection receiving antimicrobial therapy	700	Acetaminophen group vs placebo group	Early administration of acetaminophen to treat fever due to probable infection did not affect the number of ICU-free days	Young et al. (2015)
Single-center retrospective study	Mechanically ventilated septic adults	76	Lower vs higher temperature group	No differences in use of vasopressors, parameters of mechanical ventilation or survival, significantly greater use of paracetamol, esophageal cooling and acquisition of MDRP in the low temperature group	Markota et al. (2022)
Pilot RCT	Mechanically ventilated afebrile septic adults	56	Forced-air warming of critically ill afebrile patients (1.5°C above the lowest temperature documented)	External warming had lower 28-day mortality (18% vs 43%, p = )	Drewry et al. (2022)

Legend: RCT, randomized controlled trial; ICU, intensive care unit; MDRP, multiple-drug resistant pathogen.

### Therapeutic hypothermia in patients with infection

Use of hypothermia in some animal models was associated with decreased intracranial pressure and modulation of nuclear factor-κB activation (Irazuzta et al., 2002). In a small, uncontrolled, single-center clinical study published in 2011 favorable outcomes were observed after induction of hypothermia (32°C–34°C) in a group of 10 patients with severe bacterial meningitis and signs of increased intracranial pressure (Lepur et al., 2011). However, a large, multicenter, randomized trial published in 2013 in patients with severe bacterial meningitis was stopped early after 98 patients were randomized (inclusion of 276 patients was planned) because significantly higher mortality was observed in the hypothermia group (51% vs. 31%, *p* = 0.04) (Mourvillier et al., 2013). Hypothermia was induced by infusion of cold saline and temperature separation of around 4°C was achieved (approximately 33°C in the hypothermia group vs. 37°C in the control group). Of note, there were no differences between groups in new onset infections, control lumbar punctures in both groups were all negative, and there were no differences in cerebrospinal fluid leukocyte counts between both groups; however, the proportion of patients who developed septic shock was higher in the hypothermia group (47% vs. 32%) (Mourvillier et al., 2013).

Similarly, a large, multicenter, randomized trial published in 2018 was designed to detect a 21% reduction in 30-day all-cause mortality in a cohort of 560 adults (age≥50 years) with severe sepsis or septic shock (Itenov et al., 2018). The primary goal was based, among other studies, on a previous smaller trial enrolling 200 febrile patients in septic shock where external cooling to normothermia (target temperature 36.5°C–37°C) for 48 h reduced the need for vasoactive therapy compared to no external cooling. However, the larger trial was stopped early for futility after recruitment of 436 patients, with higher mortality observed in the hypothermia group (44.2% vs. 35.8°%, *p* = 0.07). Hypothermia was induced either by an intravascular device or by external cooling. Patients in the hypothermia group required higher doses of vasopressors, suffered a more severe respiratory failure and a delayed decrease of C-reactive protein. The authors hypothesized that lymphocytopenia and immune system paresis, among others, could have contributed to worse outcomes in the hypothermia group (Itenov et al., 2018).

### Paracetamol to manage fever in general ICU patient population

Fever is one of the most common indications for the application of paracetamol. In a large, randomized, multicenter trial published in 2015, 700 adults hospitalized in the ICU with body temperature ≥38°C due to probable infection were randomized to receive either paracetamol or placebo (Young et al., 2015). Temperature separation between the two groups of around 0.5°C was achieved; however, no differences were observed in the duration of ICU treatment, 28- and 90-days mortality, requirement for vasopressors, mechanical ventilation parameters or requirement for renal replacement therapy. They observed significantly shorter ICU stay in the paracetamol group in survivors (3.5 days vs. 4.3 days, *p* = 0.01), and significantly longer ICU stay in the paracetamol group in non-survivors (10.4 days vs. 4 days, *p* < 0.001), which was explained by the clinicians’ perception of illness severity and prognosis, which is affected by body temperature (Mackowiak et al., 1982). Similar results were reported in a meta-analysis of 13 trials published in 2021, comparing antipyretics with placebo in non-neurocritically ill patients (Sakkat et al., 2021).

### Greater incidence of multiple drug resistant microorganisms in patients with infection and lower temperature

In some patients physical cooling methods are utilized because of presumed benefits of fever control. There are no guidelines regarding target temperatures for general ICU patient population (i.e., mostly patients without signs of central nervous system disease), so target temperatures are determined by the treating clinicians. In a single-center retrospective study of 76 patients published in 2022 no differences in use of vasopressors, parameters of mechanical ventilation or survival were found between patients with body temperature ±0.5°C within target temperature for ≥25% of time (and lower body temperature), and patients with body temperature outside target temperature for <24% of time (and higher body temperature) (Markota et al., 2022). Significant differences were found in the use of paracetamol, esophageal cooling, and greater prevalence of multidrug resistant pathogens, which were all greater in the group of patients with ≥25% of time within target temperature range. A higher proportion of time within target range can be explained by the use of paracetamol and esophageal cooling. Greater prevalence of multidrug resistant pathogens in the group of patients who were ≥25% of time within target temperature range and had lower temperature could be in part explained by lower temperature itself, but other factors could also have contributed, e.g., probably more patient interventions were required in the group of patients who were ≥25% of time within target temperature range which could have contributed to the acquisition of multidrug resistant pathogens (Markota et al., 2022).

### Therapeutic hyperthermia in the modern era

To our knowledge only one prospective trial comparing TH to standard care has been performed in the modern era. Drewry et al. performed a small, randomized, single-center, pilot trial published in 2022 on adult patients with sepsis who did not develop fever (maximum temperature <38.3°C) (Drewry et al., 2022). Patients were randomized to two groups: in the hyperthermia group patients were warmed for 48 h to a temperature 1.5°C above the lowest temperature recorded 24 h before randomization, while patients in the control group received standard temperature management. No differences were observed in the primary (HLA-DR expression) and secondary outcomes (IFN-γ production); however, lower 28-day mortality (18% vs. 43%) and more 28-day hospital free days (difference 2.6 days) were observed in the hyperthermia group. Hyperthermia was well tolerated by the participants and warming was terminated in only two (7%) patients because predetermined increase in vasopressor dose was reached (vasopressor increase greater than 50% for 6 h and greater than 0.1 μg/kg/min of norepinephrine); however, the same safety criterion was reached by four (14%) patients in the control group. There were no differences in acquisition of hospital acquired infections (30% in both patients) (Drewry et al., 2022).

## Conclusion

While antimicrobials will continue to serve as the cornerstone of non-surgical infection treatment in the future, the increasing prevalence of multi-resistant bacteria and the emergence of viral epidemics necessitate the exploration of non-pharmacological treatment approaches. TH has been employed in the past for the management of infections. Several studies have indicated that prevention of fever in non-neurocritical patients with infection is not associated with better survival or favorable course of ICU treatment. There is evidence suggesting harm associated with lowering the body temperature, and in the most high-risk patients with sepsis (patients who do not develop a febrile response), even artificial rewarming could be reasonable. If fever develops as part of a physiological response to infection, it is probably reasonable to adopt a temperature tolerance strategy in the general population of ICU patients.
